# Does efavirenz replacement improve neurological function in treated HIV infection?

**DOI:** 10.1111/hiv.12503

**Published:** 2017-03-01

**Authors:** B Payne, TJ Chadwick, A Blamire, KN Anderson, J Parikh, J Qian, AM Hynes, J Wilkinson, DA Price, the Efficacy of Switch to Lopinavir/Ritonavir in Improving Cognitive Function in Efavirenz‐treated Patients (SLICE) study team

**Affiliations:** ^1^ Department of Infection and Tropical Medicine The Newcastle Upon Tyne Hospitals NHS Foundation Trust Newcastle UK; ^2^ Wellcome Trust Centre for Mitochondrial Research Institute of Genetic Medicine Newcastle University Newcastle UK; ^3^ Institute of Health and Society Newcastle University Newcastle UK; ^4^ Centre for In Vivo Imaging Newcastle University Newcastle UK; ^5^ Regional Sleep Service The Newcastle Upon Tyne Hospitals NHS Foundation Trust Newcastle UK; ^6^ Newcastle Clinical Trials Unit Newcastle University Newcastle UK

**Keywords:** cognitive impairment, efavirenz, functional magnetic resonance imaging, HIV, magnetic resonance spectroscopy

## Abstract

**Objectives:**

The contribution of specific antiretroviral drugs to cognitive function in HIV‐infected people remains poorly understood. Efavirenz (EFV) may plausibly cause cognitive impairment. The objective of this study was therefore to determine whether chronic EFV therapy is a modifier of neurocognitive and neurometabolic function in the setting of suppressive highly active antiretroviral therapy.

**Methods:**

We performed an open‐label phase IV controlled trial. Adult subjects who were stable on suppressive EFV therapy for at least 6 months were switched to ritonavir‐boosted lopinavir (LPV/r) with no change in the nucleoside reverse transcriptase inhibitor (NRTI) backbone. The following parameters were assessed before and 10 weeks after therapy switch: cognitive function (by CogState^®^ computerized battery); brain metabolites (by proton magnetic resonance spectroscopy); brain activity [by attentional processing task‐based functional magnetic resonance imaging]; and sleep quantity and quality [by sleep diary, Pittsburgh Sleep Quality Index (PSQI) and Epworth Sleepiness Scale].

**Results:**

Sixteen subjects completed the study. Despite most subjects (81%) self‐reporting memory problems at baseline, cognitive function, brain metabolites, and brain activity showed no change at 10 weeks after switch. Sleep quality improved on switch off EFV [mean PSQI (standard deviation): EFV, 8.5 (6.5); LPV/r, 5.8 (5.5); mean difference −0.4; 95% confidence interval −6.0 to −0.7].

**Conclusions:**

This is the first study to assess the effects of chronic EFV therapy on neurological function in a controlled setting. We conclude that EFV withdrawal is unlikely to result in significant modification of neurocognitive function in otherwise stable HIV‐infected people.

## Introduction

Mild cognitive impairment remains common in the highly active antiretroviral therapy (HAART) era, but the reasons for this remain incompletely understood 1. In particular, the role of specific antiretroviral drugs in mediating cognitive function has received relatively little attention 2. While it is essential to treat viral replication in the central nervous system (CNS), it is plausible that some antiretroviral drugs also have adverse effects on neuronal function. Efavirenz (EFV) remains one of the most commonly used antiretroviral drugs. It is well recognized that CNS side effects (especially sleep disturbance) are common in the first 6 weeks of EFV therapy and sometimes lead to discontinuation, but generally subside with continued therapy 3, 4. Some recent observational studies have suggested that EFV may additionally be associated with increased rates of cognitive impairment, a notion that is supported by some neurotoxicity data from animal and *in vitro* studies 5, 6, 7, 8, 9, 10. This hypothesis, however, remains controversial and has not been confirmed in a controlled trial.

The aim of this study was therefore to determine whether chronic EFV therapy is a modifier of neurocognitive function in the setting of suppressive HAART.

## Methods

### Participants

We performed a self‐controlled, open‐label phase IV pilot study. Participants were adult patients (aged 18–65 years) with HIV‐1 infection receiving suppressive HAART with an EFV‐containing regimen. All observations were performed at a single study site. Subjects were required to have a documented HIV‐1 RNA viral load (VL) measurement of < 50 HIV‐1 RNA copies/ml within the 4 months preceding study entry and no VL exceeding 200 copies/ml within 12 months. Subjects were on HAART for at least 12 months and on EFV for at least 6 months. This constraint was to ensure that only chronic effects of EFV were captured. Subjects were ineligible if they had current self‐reported recreational drug use or weekly alcohol consumption exceeding 35 units.

All subjects gave written informed consent for participation in the study. The study was approved by the local research ethics committee (12/NE/0071), Newcastle‐upon‐Tyne Hospitals NHS Foundation Trust R&D approvals committee (5946), and the UK regulatory authority Medicines & Healthcare products Regulatory Agency (MHRA) and registered with the EudraCT trials database (2011‐005581‐37).

### Interventions and measurements

Ritonavir‐boosted lopinavir (LPV/r; Kaletra^®^, AbbVie Inc., North Chicago, IL, USA; twice daily dosing) was used as a comparator drug to assess the effects of EFV removal. All subjects switched from EFV to LPV/r. Participants had study observations performed at baseline and 10 weeks after switch. An additional safety monitoring visit was performed 4 weeks after therapy switch.

A computerized cognitive testing battery (CogState^®^, CogState Ltd., Melbourne, Australia) was performed comprising six tests [listed with abbreviated test name; cognitive domain(s) measured]: Detection (DET; psychomotor function/speed of processing), Identification (IDN; visual attention/vigilance), One card learning (OCL; visual learning and memory), One back (ONB; attention/working memory), Continuous paired associate learning (CPAL; visual learning and memory), and Groton maze learning (GML; executive function/spatial problem solving). Subjects performed a practice run of each test to minimize training effects. Baseline cognitive impairment was defined with respect to CogState^®^ normative data (excluding CPAL as sufficient quality data are not currently available for this test) 11.

Proton magnetic resonance spectroscopy (^1^H‐MRS) was performed using PRESS (point resolved spectroscopy) volume selection (TR/TE (repetition time / echo time) = 3 s/37 ms) on a 3T magnet (Achieva; Philips Medical Systems, Amsterdam, Netherlands) with ~8 cm^3^ sampled voxels in frontal white matter (FWM), frontal grey matter (FC) and basal ganglia (BG). Metabolite levels of N‐acetylaspartate (NAA), choline (Cho) and creatine (Cre) were quantified using the QUEST method in jMRUI software 12. Concentrations were expressed relative to Cre. NAA is a widely studied measure of neuronal integrity and is frequently abnormal in HIV infection. Cho is frequently altered in inflammation.

Task‐based (attentional processing) functional magnetic resonance imaging (fMRI) assessed response to the Stroop paradigm using incongruent visual stimuli 13. The Stroop test has been shown to be sensitive to psychotropic drug effects including stimulants and sedatives 14, 15, 16, as well as to EFV use in one previous study 5. fMRI was performed using a gradient‐echo EPI (echo planar imaging) sequence (TR/TE = 1.7 s/30 ms; 27 × 4.0 mm thick slices; 0.5 mm inter‐slice gap; 3 mm in‐plane resolution) with a block design Stroop paradigm. Data were collected in a single fMRI acquisition during which 360 image volumes were acquired. The stimulus consisted of a rest period, 18 interleaved blocks of incongruent and neutral stimuli and a final rest period. Subject responses were monitored using a response box. Analysis of the fMRI scans was performed using standard processing in spm8 (http://www.fil.ion.ucl.ac.uk/spm/software/spm8/). Analysis was performed for each subject contrasting the incongruent response against the neutral response. A group analysis was then conducted using within‐subject paired comparison of responses at each time‐point.

Sleep was assessed by means of a 2‐week sleep diary, completion of the Epworth Sleepiness Scale (ESS) (where ESS > 10 indicates excessive daytime sleepiness) and completion of the Pittsburgh Sleep Quality Index (PSQI) (where PSQI > 5 indicates some level of sleep disturbance and > 10 indicates significant sleep disturbance) 17, 18.

### Statistics

As this was a pilot study, a pragmatic sample size was used 19. Descriptive statistics and confidence intervals (CIs) are reported. No data replacement or imputation of missing data took place.

## Results

Seventeen participants were recruited, of whom 16 (three female and 13 male) completed the study (one subject was withdrawn at the screening visit). The median age was 50.4 years [interquartile range (IQR) 43.2−55.7 years]. The median duration of diagnosed HIV infection was 6.7 years (IQR: 4.2−10.0 years). Subjects had been receiving EFV for a median of 4.5 years (IQR: 4.0−5.8 years). At baseline, 14 subjects were receiving a backbone of tenofovir/emtricitabine, and the remaining two subjects were receiving tenofovir/raltegravir and abacavir/lamivudine, respectively. The median CD4 lymphocyte count at entry was 660 cells/*μ*L (IQR: 536−737 cells/*μ*L), and the median nadir CD4 count was 237 cells/*μ*L (IQR: 37−299 cells/*μ*L; *n* = 12 subjects with available data for nadir). The median baseline EFV plasma concentration was 2455 ng/mL (range 818–7197 ng/mL; *n* = 15 subjects with analysable EFV levels).

Subjects frequently reported CNS symptoms at both baseline and follow‐up, although the pattern of symptoms varied between visits (*n* = 16): memory problems (81% of subjects at baseline and 31% at follow‐up), vivid/intrusive dreams (75% and 44%, respectively), fatigue (69% and 25%, respectively), concentration difficulties (63% and 75%, respectively), and sleep problems (56% and 81%, respectively).

Nine subjects (56%) showed evidence of mild cognitive impairment at baseline [defined as performance > 1 standard deviation (SD) below age‐specific norm (i.e. *z*‐score < −1) on at least two domains]. Two subjects (13%) showed severe impairment (*z*‐score < −2 on at least two domains). No change in cognitive performance was observed between baseline and follow‐up visits. Mean change score (reported such that a positive change indicates an improvement) for each task was as follows (*n* = 16): DET, −0.03 (95% CI: −0.09 to 0.04); IDN, 0.03 (95% CI: −0.02 to 0.07); OCL, 0.03 (95% CI: −0.004 to 0.07); ONB, −0.001 (95% CI: −0.07 to 0.07); CPAL, 13.6 (95% CI: −9.8 to 37.0); GML, 2.9 (95% CI: −3.5 to 9.4). Summary statistics for cognitive testing are presented in Table 1.

**Table 1:** Neurocognitive testing by the CogState^®^ battery

Task	*n*	Baseline (EFV)	Follow‐up (LPV/r)	Change
		Mean (SD)	Mean (SD)	Mean (95% CI)
DET^a^	16	2.56 (0.08)	2.59 (0.10)	−0.03 (−0.09, 0.04)
IDN^a^	16	2.78 (0.07)	2.75 (0.07)	0.03 (−0.02, 0.07)
OCL^b^	16	0.92 (0.11)	0.96 (0.11)	0.03 (−0.004, 0.07)
ONB^b^	16	1.3 (0.13)	1.3 (0.14)	−0.001 (−0.07, 0.07)
CPAL^a^	16	121 (46.6)	107.4 (60.1)	13.6 (−9.8, 37.0)
GML^a^	16	62.8 (22.4)	59.8 (24.7)	2.9 (−3.5, 9.4)

CI, confidence interval; EFV, efavirenz; LPV/r, ritonavir‐boosted lopinavir; SD, standard deviation.

Detection (DET) and Identification (IDN): speed of performance (mean of the log_10_‐transformed reaction times for correct responses). One card learning (OCL) and One back (ONB): accuracy of performance (arcsine transformation of the proportion of correct responses). Continuous paired associate learning (CPAL): accuracy of performance (total number of errors across five rounds). Groton maze learning (GML): total number of errors made on five consecutive trials at a single session. (Data for one patient for one task at one visit failed the integrity check. The analysis was performed with and without inclusion of this data point and the results were not affected.)

a A lower score indicates a better performance, and change is defined as baseline score minus follow‐up score.

b A higher score indicates a better performance, and change is defined as follow‐up score minus baseline score, so that in all cases a positive change indicates improvement.

Subjects were included in paired ^1^H‐MRS analysis where spectral quality for a given voxel was acceptable at both study time‐points, as follows: FWM, *n* = 11; FC, *n* = 14; BG, *n* = 8. No changes were observed for any metabolite in any voxel between baseline and follow‐up visits, with mean change as follows: FC: Cho/Cre, 0.01 (95% CI: −0.01 to 0.02) and NAA/Cre, 0.16 (95% CI: −0.13 to 0.44); FWM: Cho/Cre, 0.03 (95% CI: −0.02 to 0.08) and NAA/Cre, 0.11 (95% CI: −0.12 to 0.33); BG: Cho/Cre, 0.04 (95% CI: −0.02 to 0.10) and NAA/Cre, 0.03 (95% CI: −0.32 to 0.38). Summary statistics for ^1^H‐MRS are presented in Table 2.

**Table 2:** Brain metabolites measured by proton magnetic resonance spectroscopy (^1^H‐MRS)

		Baseline (EFV)			Follow‐up (LPV/r)			Change		
		*n*	Mean	SD	*n*	Mean	SD	*n*	Mean	95% CI
FC	Cho/Cre	14	0.26	0.03	14	0.27	0.03	14	0.01	−0.01, 0.02
	NAA/Cre	14	1.8	0.27	14	1.96	0.35	14	0.16	−0.13, 0.44
FWM	Cho/Cre	13	0.25	0.05	11	0.3	0.07	11	0.03	−0.02, 0.08
	NAA/Cre	13	1.38	0.33	11	1.51	0.22	11	0.11	−0.12, 0.33
BG	Cho/Cre	9	0.22	0.09	9	0.23	0.04	8	0.04	−0.02, 0.10
	NAA/Cre	9	2.34	0.78	9	2.13	0.31	8	0.03	−0.32, 0.38

FC, frontal cortex (frontal grey matter); FWM, frontal white matter; BG, basal ganglia; Cho, choline; Cre, creatine; NAA, N‐acetylaspartate; CI, confidence interval; EFV, efavirenz; LPV/r, ritonavir‐boosted lopinavir; SD, standard deviation.

fMRI data for 14 subjects were analysable. First, the effects of the ‘Stroop’ task on brain activity were assessed at the baseline visit to ensure that the fMRI protocol employed elicited a measureable and expected effect in our subject group. The analysis assessed activity during the incongruent stimulus, contrasted against that during the neutral stimulus. Three clusters of activation during the task were identified, in an anatomical distribution corresponding to that expected for this test paradigm: BA45/BA8/BA46, BA20/BA7/BA40 and BA7 (BA, Brodmann's area; Table S1). Secondly, a comparison was made of brain activation in response to the incongruent stimulus between the two study time‐points. No change in brain activation was observed following switch.

There was no overall change in the ESS for patients on a switch from EFV to LPV/r [mean ESS (SD): EFV, 9.8 (5.9); LPV/r, 8.9 (5.1); mean difference −0.9; 95% CI: −2.7 to 0.9]. Sleep diary responses were used to calculate mean hours of sleep per subject per 24‐h period. Fifteen subjects were included in this analysis. Mean change in mean hours of sleep between baseline and follow‐up visits was 0.1 h (95% C: −0.7 to 1.0 h). Change in sleep quality (PSQI) was analysable for 14 subjects. The mean score decreased, indicating an improvement in sleep quality [mean (SD): EFV, 8.5 (6.5); LPV/r, 5.8 (5.5); mean difference −3.4; 95% CI: −6.0 to −0.7]. Of seven patients with a PSQI > 10 (indicating poor sleep quality) at baseline, four patients had a PSQI of < 10 at follow‐up. No subjects had an insomnia phenotype (low ESS < 2 with high PSQI > 10) at either time‐point. As executive function is most typically affected by sleep disturbance, we assessed the data for any relationship between baseline sleep quality and GML score. Mean GML score did not differ in subjects with an abnormal PSQI score (> 10). Furthermore, there was no association between baseline EFV plasma concentration and baseline GML, ESS or PSQI scores or change in these scores after cessation of EFV.

HIV‐1 RNA VL was checked at the safety monitoring visit 4 weeks after treatment switch. At this point, three subjects (19%) had VL > 50 copies/mL, with values of 69, 107 and 115 copies/mL, respectively. By the follow‐up visit, the first two subjects had re‐suppressed to < 50 copies/mL, whereas the third subject had a VL of 337 copies/mL. Self‐reported adherence was assessed at each of three follow‐up visits, and at each visit four subjects reported at least one missed dose per week. At baseline, three subjects (19%) reported diarrhoea (of any severity). Fourteen subjects reported diarrhoea at any time following the switch from EFV to LPV/r (88%) (11 of the 13 not affected at baseline; 85%). There were no serious adverse events (SAEs) recorded.

## Discussion

We have explored the hypothesis that chronic EFV therapy is associated with adverse effects on CNS function. To our knowledge, this is the first study to assess this question in a controlled manner by investigating the effects of a switch away from EFV on neurocognitive performance, cerebral metabolites, brain activity, and sleep. Despite the fact that a very high proportion of subjects self‐reported CNS symptoms at baseline (including 81% reporting memory problems), we observed no objective changes in neurocognitive performance across multiple domains, in cerebral metabolites, or in brain activity. There was, however, an increase in self‐reported sleep quality (PSQI).

This was a pilot study, and so our ability to detect small effect sizes will have been limited by the sample size. Notwithstanding this, for almost all parameters studied, the effect sizes observed were very close to zero, and there were no consistent trends in directions of change across related domains (for example in cerebral metabolites or cognitive performance). We therefore conclude that we are unlikely to have missed any clinically important effects.

Given that previous studies have suggested adverse effects of EFV on neurocognitive function, what are the possible explanations for our findings? As previous studies associating cognitive impairment and EFV were observational and cross‐sectional in nature, they were liable to being confounded by differences between groups exposed and unexposed to EFV 5. In the one randomized controlled trial (RCT) that has compared EFV and a protease inhibitor, the study was performed in the setting of treatment‐naïve subjects starting HAART 20. Thus, the predominant effect was changes in cerebral metabolites associated with suppression of viral replication and immune reconstitution, and differences observed between drugs may not have been wholly independent of their effects on those parameters. Although most of our subjects did report at least some CNS symptoms at baseline, those patients who experience severe early neuropsychiatric side effects of EFV are likely to switch away from the drug within the first few months and therefore would not be represented in our study. Furthermore, previous EFV switch studies have documented improvement in CNS symptomatology, but have not included objective measures of CNS performance 21, 22. In theory, a switch off EFV might have prevented a decline in cognitive function that would otherwise have occurred, but this seems very unlikely given the short study period. Alternatively, if LPV/r were slightly less effective at suppressing viral replication in the CNS, this could potentially offset any benefits from EFV withdrawal. We did note some VL blips and reported missed doses after treatment switch. Again, it seems unlikely that this would affect CNS function within the timeframe studied, but this should be explored in future studies by correlation with cerebrospinal fluid (CSF) HIV VL. A final possibility is that long‐term EFV therapy could cause a mild but permanent deficit in CNS function (e.g. as a result of neuronal loss) which would not be reversible following therapy switch. We did observe around half of subjects with mild cognitive impairment at baseline, but this study cannot determine whether this was attributable to EFV or other factors. This question could only be explored by a very long follow‐up RCT.

In summary, our study did not find support for an acutely reversible effect of EFV on either CNS metabolites or cognitive function in otherwise stable patients. These findings should be confirmed by further randomized studies.

## Supporting information


**Data S1**: Areas of brain activation on fMRI.
