# Supplementary material for: Does efavirenz replacement improve neurological function in treated HIV infection?
Source: HIV Med. 2017 Mar 1;18(9):690–5. doi: 10.1111/hiv.12503 (PMC5600135; doi:10.1111/hiv.12503)
Supplement: Supplementary file 1 — Data S1: Areas of brain activation on fMRI. [file HIV-18-690-s001.docx]

***Does efavirenz replacement improve neurological function in treated HIV infection?***

**Supplementary Data: Areas of brain activation on fMRI.** Effects of Stroop test incongruent compared with neutral stimulus at baseline visit.

Notes: BA, anatomical Brodmann area. Analysis was performed using SPM8 software. 14 subjects were included in the analysis (2 subjects were omitted due to registration errors (it was not possible to match the subject data to the standard brain template)).

| **Coordinates**  **(x y z mm)** | **Extent (Pixels)** | **P value (cluster)** | **Peak T** | | **Brain Region** | |
| --- | --- | --- | --- | --- | --- | --- |
| -42 17 16  -48 11 40  -45 32 16 | 156 | <0.001 | | 6.78  4.45  4.32 | | Left Inferior Frontal Gyrus(BA 45)  Left Middle Frontal Gyrus (BA 8)  Left Middle Frontal Gyrus (BA 46) |
| -33 -6 46  -30 -57 52  -45 -52 52 | 136 | <0.001 | | 6.14  5.82  5.52 | | Left Inferior Temporal Gyrus (BA 20)  Left Superior Parietal Lobe (BA 7)  Left Inferior Parietal Lobe (BA 40) |
| 36 -67 46 | 25 | 0.028 | | 5.25 | | Right Superior Parietal Lobe (BA 7) |


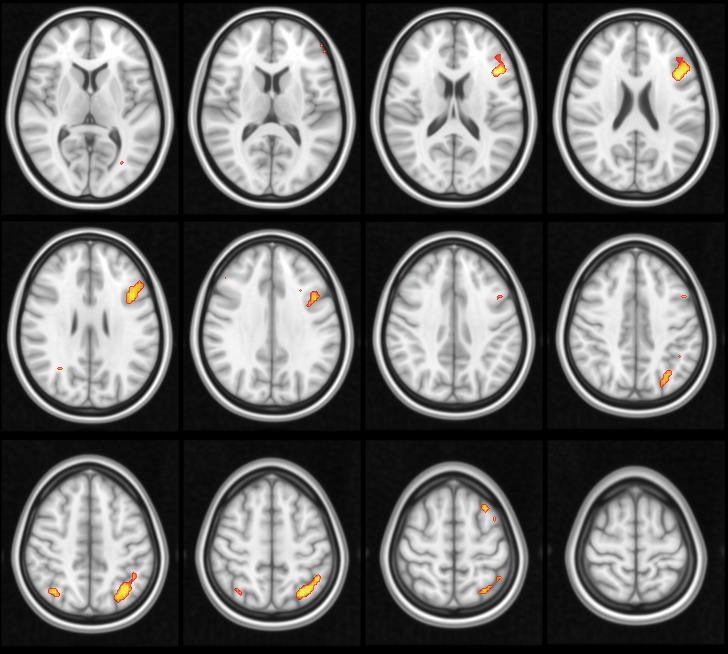


Heatmap of areas of brain activation.
